# Using Surveys to Calculate Disability-Adjusted Life-Years

**DOI:** 10.35946/arcr.v35.2.03

**Published:** 2014

**Authors:** Wolfgang Wiedermann, Ulrich Frick

**Affiliations:** **Wolfgang Wiedermann, Ph.D.,***is a junior researcher at the Carinthia University of Applied Sciences, Feldkirchen, Austria.*; **Ulrich Frick, Ph.D.,***is a professor of public health at the Carinthia University of Applied Sciences, Feldkirchen, Austria, and senior scientist at the Psychiatric University Hospital, University of Regensburg, Regensburg, Germany.*

## Abstract

Mapping a certain disease into a system of disabling attributes allows researchers to compare diseases within a common framework. To quantify the total burden of morbidity (e.g., morbidity attributable to alcohol use), so-called disability weights (DWs) must be generated. General-population surveys can be used to derive DWs from health valuation tasks. This article describes the application of three psychometric methods (i.e., pairwise comparisons, ranking tasks, and visual analog scales) in general-population surveys and outlines their strengths and weaknesses. A recently proposed health valuation framework also is presented, which highlights the underlying cognitive processes from a social-judgment perspective and presents a structured data-collection procedure that seems promising in deriving DWs from general-population surveys.

To quantify the burden of a disease within a population, a health-gap measure is more useful than measures of health expectancy or quality-adjusted life-years (see Etches et al. 2006). Disability-adjusted life-years (DALYs), the most prominent of the health-gap measures, combine the burden attributable to early death and to morbidity into one single number. Alcohol affects a long list of diseases and disabilities in varying intensities, each of which can be described by a number of health-state attributes. Common measures of health outcomes include the EuroQol5D (EQ5D) (Brooks and EuroQol Group 1996), the Health Utilities Index III (HUI III) (Feeny et al. 2002), the Short-Form 36 Health Survey (SF36) (Ware and Sherbourne 1992), and the CLAssification and MEasurement System of Functional Health (CLAMES) (McIntosh et al. 2007). Mapping a certain disease into a system of disabling attributes (e.g., physical functioning, pain, memory and thinking, etc.) enables health researchers to compare qualitatively different diseases within a common framework. To quantify the total burden of alcohol-attributable morbidity, it is necessary to provide so-called disability weights (DWs) for each of these health states, which are bounded by the DWs of 0 (for complete health) and 1 (for death). It should be noted that health states are considered, rather than diseases with labels (and their psychological and/or medical implications), when DWs are determined.

How DWs can validly be measured, defined, or (more neutrally speaking) elicited is of equal importance for the results as the question, “Who is asked to provide the DWs?” Although elicitation methods will be discussed below, this article does not focus on the question of which sources (e.g., patients, clinical experts, etc.) should be consulted to quantify DWs. Rather, this article considers only general-population surveys (i.e., telephone, face to face, or mailed) as sources of information on the disabilities associated with different health states.

## How Are DWs Elicited?

Three popular methods to construct DWs stem from econometric utility theory: standard gamble (SG), time tradeoff (TTO), and person tradeoff (PTO). They all share the central idea that a respondent’s point of indifference, at which he or she cannot unequivocally decide on a certain judgmental task, enables researchers to measure utility differences via the traded “goods.” For example, in SG, respondents are given a choice between an outcome that is certain (i.e., remaining in ill health) and a gamble with one better and one worse outcome (e.g., full health or death). Respondents are asked what probability of the better outcome would make them indifferent to remaining in the described state (ill health) for certain or choosing the risky option. Therefore, if they are indifferent to the ill-health state and gamble with a 0.8 probability of the better outcome (but 0.2 probability of the worse outcome), 0.8 represents the utility of the ill health.

In a TTO task, respondents are asked to consider the relative amounts of time (e.g., number of life-years) they would be willing to sacrifice to avoid a certain poorer health state (e.g., frequent headaches). Assuming a scenario of 10 years with frequent headaches, the respondent may be indifferent to this state and a shorter lifetime of 7 years, resulting in an estimated utility for the frequent-headaches health state of 0.7 (7 years divided by 10 years).

A typical PTO elicitation asks respondents to choose between two equally expensive health care treatment programs that improve quality of life or save lives for two groups of patients. The decisionmaker must choose to fund one of the two mutually exclusive programs, one of which has a fixed number of patients. Respondents are asked how many patients would need to be treated to make them indifferent to the two programs. For example, program A might extend the life of 100 healthy individuals for 1 year, whereas program B might cure 100 individuals of a chronic health condition.

All three methods are time consuming, require highly motivated respondents, and are hardly feasible without a trained interviewer or computer program. Whereas TTO has been used in face-to-face interviews in the general population quite often (e.g., Badia et al. 2001; Chevalier and de Pouvourville 2011; Dolan 1997; Greiner et al. 2005; Jelsma et al. 2003; Jo et al. 2008; Lamers et al. 2006; Lee et al. 2009; Shaw et al. 2010; Tsuchiya et al. 2002; Wittrup-Jensen et al. 2009; Zarate et al. 2008), in mail surveys only two studies (Burström et al. 2006; Lundberg et al. 1999) used TTO to quantify respondents’ own health states. SG and PTO have rarely been used for eliciting health-state preferences in mailed surveys (i.e., they are usually used in face-to-face or phone interviews), and they have only been used among former patients (i.e., not the general public) (Hammerschmidt et al. 2004).

Readers are referred to Rehm and Frick (2010) for an overview on the methodological problems associated with econometric elicitation methods in this context. Recently, Wittenberg and Prosser (2011) described two additional sources of bias or mistaken responses in preference measurement in surveys: ordering errors (i.e., illogical responses, which violate a naturally given order, whereas inconsistent responses contradict each other within a person), and objections/invariance (i.e., respondents may refuse to participate because of an unwillingness to trade time [in the TTO task] or risk [in the SG task]). Furthermore, the meaning of SG results has been criticized as rather a measurement of risk attitude than a representation of subjective utility (Lenert and Kaplan 2000). TTO results as a metric for utility have been shown to vary with respondents’ age, education, and current health state (Ayalon and King-Kallimanis 2010; Meropol et al. 2008; Stiggelbout et al. 1996; Voogt et al. 2005). Feasibility of PTO frequently is hampered because people tend to refuse such tasks because of their desire to avoid prejudice and discrimination (Damschroder et al. 2005).

As alternatives to the methods described above, psychometric theory provides paired comparisons, ranking tasks, and visual analogue scales as tools to elicit health-state preferences. These tools are discussed below.

## Paired Comparisons

In the context of health-state valuation, a paired comparison (PC) task simply means that respondents must choose which of two given states is more disabling, worse, or dominant in some way. Because measuring via PC seems quite simple and feasible (because it is only necessary to present all health states in a consistent descriptive system), it has been applied in various surveys in the general population (Bijlenga et al. 2009; Kind 1982, 2005; Prieto and Alonso 2000; Ratcliffe et al. 2009; Stolk et al. 2010). For a recent application of PCs among an expert panel see Rehm and Frick (2013). Deriving DWs from the resulting pattern of dominance relations, by contrast, constitutes a complex statistical task for which solutions have been formulated from the theory of Thurstone scaling (Thurstone 1927), conditional logistic regression (Hosmer and Lemeshow 2000), and loglinear modeling (Critchlow and Fligner 1991).

Methodological challenges associated with PC stem from logically inconsistent judgments (e.g., A > B and B > C, but C > A) and from rapidly increasing burden of task when comparing larger numbers of health states (i.e., combinatorial explosion). Intransitive judgments (e.g., in comparing 10, 7, and 5, 10 is preferred to 7 and 7 is preferred to 5, but 5 is preferred to 10) may originate in unintended framing effects as well as in imperfect judgment (von Winterfeldt and Edwards 1986). Recently published experimental studies favor the position that excluding inconsistent ratings cannot improve the description of true preferences and therefore might to some degree be an inevitable consequence of the decisionmaking process itself (Linares 2009). To keep the number of judgments at manageable dimensions, several studies have used incomplete factorial designs (Bijlenga et al. 2009; Prieto and Alonso 2000; Ratcliffe et al. 2009).

Asking subjects to rank order several health states, but statistically analyzing rankings as PCs, was used as an alternative in several studies (Krabbe 2008; Ip et al. 2004). Rankings can be transformed into a series of PCs (Francis et al. 2002), which at first glance avoids inconsistent judgments.

## Ranking Tasks

Health-state rankings (i.e., putting several health states into an ordinal sequence of disability), which also provide comparative information, require less cognitive effort for survey respondents. Furthermore, simultaneous comparisons of multiple health states might be less sensitive to biases (e.g., those provoked by arbitrarily labeled endpoints of rating scales) (Maydeu-Olivares and Böckenholt 2008). Although ranking exercises had been included in numerous valuation studies as an external comparison measure for TTO and SG, researchers had not used the resulting ordinal data (McCabe et al. 2006) for construction of DWs before the seminal article by Salomon (2003). Cardinal utilities derived from health-state rankings displayed high agreement to utilities from TTO or SG methods (Craig et al. 2009*a*, *b*; Kind 2005) and were more stable in a cross-cultural comparison than weights derived from SG (Ferreira et al. 2011).

From a more theoretical viewpoint, articles by Flynn and colleagues (2010) and Flynn (2010) have raised serious statistical concerns about the use of ranks as a substitution for econometric valuation tasks. Their critique focuses on modeling assumptions and thus seems beyond the scope of this article. Nevertheless, their argument suggests that it can be important to restrict the number of alternatives to be ranked and to pay special attention to how a respondent generates rankings. In addition, Lenert and colleagues (1998) have demonstrated that reported utilities are heavily influenced by the search process used to form a certain judgment. This matches the notion that preferences often are constructed (instead of merely obtained) in the elicitation process (Slovic 1995). Ranking tasks within self-administered questionnaires might be hampered by limited control of the mechanism respondents use to generate the rank order. This introduces at least two issues: First, it remains unclear which reference attributes the respondent uses to generate the rank order, which constricts intersubjective comparability and provokes primacy biases (i.e., the tendency to give more attention to items listed first) (Bowling 2005). Second, from a more technical perspective, statistical ranking models (such as the rank-ordered logit model) assume that rankings were obtained using a particular psychological mechanism (Flynn 2010). For free rankings, however, it remains unclear which statistical model is most appropriate to describe the ranking mechanism. Furthermore, it cannot be ensured that respondents using a self-administered questionnaire judge along repeated best/worst choices, a “ping-pong” method that was shown to produce reliable data (Louviere at el. 2008).

## Visual Analog Scale

To use a visual analog scale (VAS), respondents are asked to specify their level of agreement to a statement by indicating a position along a continuous line between two endpoints. Numerous studies have used VAS responses to derive health-state values in the general population (Björk and Norinder 1999; Cleemput 2010; Devlin et al. 2003; Dolan and Kind 1996; Essink-Bot et al. 1993; Greiner et al. 2003; Johnson and Pickard 2000; Johnson et al. 1998; Leidl and Reitmeir 2011). Krabbe and colleagues (2007) proposed a methodology based on differences in VAS values, where the ranks of pairwise VAS differences are used in a multidimensional scaling analysis to estimate cardinal health-state values. However, other researchers have questioned the validity of VAS data as cardinal values (Bleichrodt and Johannesson 1997; Devlin et al. 2004; van Osch and Stiggelbout 2005) for various reasons. First, VAS tasks in which the top and the bottom endpoints are precisely defined (e.g., death versus perfect health) allow direct comparison between individuals, whereas vague labels such as “worst imaginable” and “best imaginable” hamper an interindividual comparison (Torrance et al. 2001). Second, VAS responses might be affected by a so-called end-aversion bias, the phenomenon of respondents tending to be reluctant to mark positions near the endpoints of the scale (Bleichrodt and Johannesson 1997; Robinson et al. 2001; Torrance et al. 2001). Third, a VAS score for a certain health state may depend on other states presented at the same time (i.e., context bias) (Torrance et al. 2001). Fourth, the accuracy of VAS responses may be influenced by hand preferences and which hand was used (McKechnie and Brodie 2008). Finally, the orientation of the VAS scale (vertical versus horizontal) itself might affect the shape of the resulting score distribution (e.g., Lundqvist et al. 2009). Taken together, VAS responses therefore should be interpreted on an ordinal scale level only.

## Health Valuation: A Social Judgment Perspective

Stiggelbout and de Vogel-Voogt (2008) presented a four-step framework describing respondents’ cognitive processes while valuing health states: perspective/perception of the stimulus, interpretation, judgment, and formation of a manifest response (see also Rehm and Frick 2010). For each step, several mechanisms have been identified, which may affect the final response.

### 1. Perspective/perception of the stimulus

In a meta-analysis, Dolders and colleagues (2006) reported no significant differences in preferences when patient surveys were compared with those of the general public, whereas a more recent and more extensive meta-analysis by Peeters and Stiggelbout (2010) suggests that patients differ from the general public in their valuations. Frick and colleagues (2012) reported on the importance of social relationships as determinants of health valuation, especially for health professionals. Health states hampering social relationships are judged as more disabling. Ubel and colleagues (2003) described several factors that may contribute to these discrepancies: adaptation effects (i.e., affected patients often adapt physically and emotionally to their health state, resulting in a more positive valuation of the respective state), focusing illusion (i.e., healthy people focus on impaired attributes, largely ignoring unchanged attributes of a certain disease), and contrast effects (i.e., severely ill patients may underestimate the impact of lenient diseases, while healthy people may overestimate this impact). Conducting a survey in the general public will result in a weighted mixture of affected and healthy valuation perspective.

### 2. Interpretation/primary appraisal

The interpretation of a health state depends on a subject’s values, goals, and beliefs, as well as on the cognitive framing (Kahneman and Tversky 1984) and/or context (Schwarz 1999) of the health-state description.

### 3. Judgments on health states

Like human judgments in general, these are not formed to fulfill the criteria of an exhaustive information processing. By contrast, they serve as decision rules to govern behavior (e.g., giving an answer in a questionnaire) and follow the principles of parsimony and functional pragmatism rather than coherence and rationality. Stiggelbout and de Vogel-Voogt (2008) identified various sources of biases that might be relevant in the context of health valuation, such as focusing illusion (see step 1), status quo bias (i.e., respondents are more sensitive to changes in their own health state compared with imagined health states), loss aversion (see Tversky and Kahneman 1992), or failure to anticipate negative events (i.e., poor hedonic forecasting). In addition, affects and mood are known to be highly influential during judgmental processing.

### 4. A deliberate editing of the response

In this last step, for example, a respondent’s attempt to be compatible with perceived norms (e.g., perceived fairness, political correctness, or ethical considerations) further biases a subjective valuation (Rehm and Frick 2010).

## Conclusion

Econometric elicitation methods were not originally developed for self-administered questionnaires. Given the many methodological risks of using this data collection mode, TTO, PTO, or SG elicitation methods are not recommended for paper-and-pencil surveys. Understanding the introductory scenarios and autonomously and successively approaching the point of indifference seems too complicated a task for lay respondents. Though VAS scales were developed specifically for self-administered questionnaires, their validity and reliability are too weak to measure the utilities of complex health states on the interval level. Choosing between rankings and PC tasks would mean a tradeoff between economy and validity of the measurement procedure.

Among PCs presented to respondents from the general public, those with the following characteristics seem to be most promising: (1) The number of pairs of health states should be limited (to a number determined by pre-analysis) so that annoyance effects or reactance can mostly be precluded. (2) Cognitive complexity of the health state descriptions should not exceed seven (plus or minus two) judgmental attributes (Miller 1956). However, this does not necessarily mean that health-state descriptions should be limited to seven dimensions or attributes, as respondents tend to organize redundant information into broader superconcepts. That being said, this ability should also be evaluated prior to the survey. Applying these principles would allow surveys to pose complex vignettes to respondents. (3) To avoid biases due to the direction of a comparison (e.g., A versus B is not the same as B versus A) (Wänke 1996), presentation of health states within one comparison should be randomly balanced. To avoid order effects or carryover effects, factorial design techniques that also preclude repetitive presentations of certain health states (A versus B followed by C versus D and not by A versus C, for instance) should be used in the assignment of comparison tasks to respondents. Complex survey designs like the one proposed here require adequate techniques for statistical analysis (Hox et al. 1991).
